# Comparative Study of Qualitative and Quantitative Analyses of Contrast-Enhanced Ultrasound and the Diagnostic Value of B-Mode and Color Doppler for Common Benign Tumors in the Parotid Gland

**DOI:** 10.3389/fonc.2021.669542

**Published:** 2021-07-07

**Authors:** Meiying Yan, Dong Xu, Liyu Chen, Lingyan Zhou

**Affiliations:** ^1^ Department of Radiology (Ultrasound), Cancer Hospital of the University of Chinese Academy of Sciences (Zhejiang Cancer Hospital), Institute of Cancer and Basic Medicine (IBMC), Chinese Academy of Sciences, Hangzhou, China; ^2^ Key Laboratory of Head & Neck Cancer Translational Research of Zhejiang Province, Hangzhou, China

**Keywords:** contrast-enhanced ultrasound, diagnosis, qualitative, quantitative, parotid gland

## Abstract

**Purpose:**

To preliminarily identify three common benign parotid gland tumors: pleomorphic adenomas (PA), Warthin tumors (WT), and basal cell adenomas (BCA) by qualitative and quantitative analyses using contrast-enhanced ultrasound (CEUS).

**Methods:**

Preoperative images of parotid gland masses were analyzed, including 129 cases of ultrasonography (US) and color Doppler sonography (CDS) and 110 cases of qualitative and quantitative CEUS. The diagnosis was confirmed by postsurgical pathology outcomes.

**Results:**

PA presented low and heterogeneous enhancement and echo-free area, whereas most WT and BCA presented with high and relatively homogeneous enhancement. Compared with WT and BCA groups, a “slow in” pattern was more common in the PA group and a “slow out” pattern was more frequently noted in the WT group than in the PA and BCA groups. The unique features of qualitative CEUS in the PA group enable distinguishing PA from the 2 other groups. The further distinction among the groups was made based on quantitative parameters of time-intensity curves (TICs), which revealed that the mean peak intensity (PI), mean transit time (MTT), the area under the curve (AUC), and time from peak to one half (HT) exhibited significant differences. ROC analysis was next applied to determine the optimal cutoff points to predict the diagnostic tendency among the groups. When the rising slope (RS) was >2.145, the possibility of BCA was greater than WT.

**Conclusions:**

CEUS ultrasound is of significant value in the differential diagnosis of the 3 common benign parotid gland masses.

## Introduction

Tumors of the salivary gland are relatively rare, constituting a mere 5.0% of all head and neck tumors. Parotid gland tumors account for approximately 80.0% of all salivary gland tumors, most of which (75.0–80.0%) are benign (1). Pleomorphic adenomas (PA) are the most common tumor of all parotid gland tumors, which are also benign mixed tumors accounting for approximately 60.0% of all parotid tumors. As its name indicates, it is a mixture of epithelium and mesenchymal components, with changes in the matrix and epithelial morphology and microstructure (2). Monomorphic adenoma (MA), unlike PA, is a type of benign parotid gland tumor that lacks the stromal cell line and is only composed of epithelial or myoepithelial components, including Warthin tumor (WT), basal cell adenoma (BCA), and oncocytoma (3). WT is the second-most common benign tumor of the parotid gland, also known as papillary cystadenoma lymphomatosum or adenolymphoma (4). The occurrence of BCA is not that common, albeit it is the third-most common type of benign tumor representing approximately 1.0–3.0% of all salivary gland neoplasms (5). Although the current recommended treatment for benign tumors in the parotid gland remains surgery, the choice of operation time and surgical approaches differ with the histological types. In 2017, the recent Fourth World Health Organization (WHO) classification listed 11 different types of benign epithelial salivary tumors. PA has a higher tendency to become malignant relative to BCA and WT, which share a much lesser degree, whereas the other 8 types never develop malignancy (6, 7).

Some past researches have described the B-Scan sonographic appearances of lesions in the parotid gland. PA is expected to present features including irregular tumors with well-defined borders and slightly heterogeneous and generally hypovascular in nature. WT has also been shown to be well-defined and hypoechoic with hypervascularization (4). However, PA can present with cystic changes and intratumoral hemorrhage in larger masses. WT can also appear as an atypical and mildly heterogeneous tumor similar to PA. The morphological features of these 2 tumors mainly rely on the composition of their tissue components, such as epithelial and myoepithelial cells of cystic and solid tissues (8). For the remaining benign entities, such as BCA, such detailed criteria have not been described in the literature. These may often be characterized by a homogeneous hypoechoic tissue texture. Beyond these points, low-grade malignancies are often difficult to differentiate from benign tumors by ultrasound (US) alone or any other imaging tool available to date. Several past studies have reported that the definitions of margin and echogenicity are not a reliable standard for the differential diagnosis of malignant and benign lesions (9, 10). Compared with the other diagnostic tools available, such as CT and MRI, the US is a cheap, effective, and safe tool. It has been reported that this technique can adequately describe 95% of common salivary gland lesions; thus, it is a widely used method for the evaluation of superficial salivary tumors (11, 12). By assessing the perfusion pattern in color Doppler sonography (CDS), even small lesions in the parotid gland tissues can be identified (8). However, although the conventional US is highly sensitive, its specificity for distinguishing between salivary gland lesions is low (11, 12).

Fine needle aspiration cytology (FNAC) is widely used in the pathological diagnosis of salivary gland lesions. However, several past studies have emphasized its limitations, such as the high incidence of false-negative results (13). Core needle biopsy is a method that can obtain sufficient tissue samples from the tumor for subsequent histological and immunohistochemical studies (14); nevertheless, the process may lead to some complications such as localized tumor dissemination (15). Moreover, salivary gland tumors are characterized by an overlap of several histopathological and microscopic features, and their biological behavior in individual cases is difficult to predict (16). Therefore, the qualitative diagnosis of parotid gland masses is challenging, and new methods need to be explored for the same.

In recent years, with the development of novel technologies and modes of evaluation, contrast-enhanced US (CEUS) can provide additional information. The advantage of CEUS is that it is nonradiative and real-time. It is a cheap method that can be reused, unlike methods like nuclear medicine, CT, and MRI. In addition, the contrast agent (SonoVue) microbubbles can accurately display the microvessels. It is a blood pool imaging agent that does not easily leak into the interstitial space (17–19). Moreover, the echo-free areas can be described *via* CEUS, and measurable and comparable perfusion kinetics can also be provided. Furthermore, this technique can provide quantitative information for diagnosis (20). Considering the richness and complexity of the vascular system in the salivary glands, CDS and CEUS can reveal the macro-and microvessels of salivary gland tumors (20, 21).

Only a few studies are available in the literature on the examination of benign parotid gland masses by CEUS (22, 23). In our study, the information pertaining to conventional US and CDS were explored to acquire preliminary diagnostic data. A comparative study of qualitative and quantitative analyses by CEUS was retrospectively reviewed in the present study to obtain more information to distinguish among the 3 common benign tumors of the parotid gland.

## Materials and Methods

### Patients

Patients’ information from our database in the Zhejiang Cancer Hospital was reviewed retrospectively from January 2017 to December 2019 for the diagnosis of benign tumors of the salivary gland. The requirement of informed consent was waived off for this study. The patient data were analyzed anonymously. All personal details were removed from the final results. The study was approved by the hospital ethics committee and was conducted in line with the Declaration of Helsinki. All patients underwent conventional US, CDS, and qualitative and quantitative CEUS examinations. The final postoperative pathological diagnosis was performed to determine the benign or malignant nature of the mass and its specific pathological type.

### Instruments and Methods

US examination was performed using the Philips iU22 (ROYAL PHILIPS; Amsterdam, the Netherlands) US system. The probe models included L12-5 (5–12 MHz) for the conventional US and L9-3 (3–9 MHz) for the CEUS examination. The gray image of the target nodule in the largest long-axis cross-section was available. A real-time CEUS examination was performed with a 7.5e15-MHz linear-array transducer (Philips iU22 xMATRIX system) that offered a good resolution for superficial soft tissues. Sulfur hexafluoride (SonoVue; Bracco, Milan, Italy) was applied as the contrast agent. SonoVue (25 mg, lyophilized powder) and normal saline (5 mL) were prepared in the form of a suspension and vibrated uniformly.

The patient was placed in a supine position, with the neck fully exposed. Conventional US scanning was employed to scan the parotid gland masses in sagittal and cross-sections to acquire complete images of the lesions and the adjacent normal tissues. The US features such as shape, size, boundary, blood flow, and the echo of the mass were observed. The contrast mode was then turned on to select the section that can clearly demonstrate the characteristics of the tumor. The probe was fixed, and the patient was instructed to relax. According to the instructions, 2.4 mL of SonoVue contrast agent was injected into the central vein of the elbow and then washed with 5 mL of saline. The entire imaging process lasted for about 120 s. The diffusion of the contrast agent into the lesion was observed, and the image was saved.

Color Doppler flow imaging (CDFI) was classified according to Adler’s method (24): Grade 0 showed no blood flow signal in the mass; Grade I showed a small amount of blood flow, with 1–2 point-like or rod-like tumor blood vessels; Grade II showed a medium amount of blood flow, with 3–4 point-like blood vessels or a single long blood vessel (close to or exceeding the mass-radius in length) penetrating the lesion; and Grade III showed rich blood flow, with ≥5 punctate vessels or two long vessels.

#### Qualitative Analysis of the CEUS Image

We retrospectively reviewed the dynamic images stored in the computer and then used them to analyze the following aspects: (1) enhancement intensity: the signal intensity of the enhancement, which was classified as high or low (all comparisons made with enhancement intensity of the normal parotid tissues at the same level); (2) the texture of enhancement: the enhancement distribution of the parotid gland masses, which was classified as homogeneous or heterogeneous; (3) margin after enhancement: after the enhancement, the margin of the mass was classified as clear or unclear; (4) ring enhancement: the appearance of a high-brightness ring enhancement around the mass, which was either a yes or no; (5) echo-free area: the appearance of a contrast-free perfusion area in the tumor after the enhancement, which was either a yes or no; (6) size of the enhanced lesions: the size of the enhanced lesions after the enhancement, which was classified as larger or similar; (7) wash-in pattern: the way of the SonoVue sign from the border of the mass into the center concentration to the peak value; and (8) wash-out pattern: the way of decreasing signal intensity of the SonoVue sign. By observing and recording the CEUS features of the lesions, the recorded characteristics included enhancement patterns: fast in, slow in, fast out, or slow out, when compared with the enhancement time of the normal parotid gland tissues.

#### Quantitative Analysis of the CEUS Image

The dynamic-enhanced images were randomly stored in the hard disk of the machines in the DICOM format and fed into the random QLAB quantitative analysis software for region-of-interest analysis (ROI). The free mode of ROI was selected both in the solid portion of the nodule and in the surrounding glandular parenchyma, with caution to avoid the cystic area, including the large vessels and necrotic areas. Subsequently, the entire mass was traced, and the time-intensity curve (TIC) was obtained. Motion correction (respiratory motion compensation) was selectively performed to accurately reflect the results. The following observational perfusion parameters were included: (1) rise time (RT, in seconds), representing the time during which the curve increases from the starting point to 50% of the peak value; (2) peak intensity (PI, in dB), representing the maximum signal intensity measured in the selected ROI; (3) mean transit time (MTT, in seconds), representing the time during which the curve decreases from the starting point to 50% of the PI; (4) area under the curve (AUC): in seconds (DB × s)/1000, representing the area under the entire time intensity curve; (5) time from peak to half (HT, in seconds), representing the time from peak to half of the absolute increment; (6) time to peak (TTP, in seconds), which is defined as the time from the beginning of the curve to PI; and (7) rising slope (RS, in dB/s), which is calculated using the formula (peak intensity−baseline intensity)/rise time.

The results of conventional US, CDS, and qualitative and quantitative CEUS were interpreted by a dedicated head and neck radiologist and two dedicated radiologists in consensus, who have been practicing in the field for 25 years, 21years, and 19 years, respectively. They were blinded to the final pathology outcomes.

### Statistical Analyses

Categorical variables were shown as percent, and statistical analysis was performed using the two-tailed χ^2^ test or Fisher’s exact test to evaluate the differences in the frequencies of the outcomes. Continuous variables were presented as mean. Since the values of different parameters did not demonstrate a normal distribution, the non-parametric Kruskal–Wallis test was employed for the statistical analysis of the age and tumor size to compare the presence of differences among the 3 groups as a whole, and Mann–Whitney U-test was used for pairwise comparison. Due to the pairwise comparison (*post hoc* test), the significance level needed to be adjusted (adjusted α Level) as the significance level of pairwise comparison. According to Bonferroni’s method, adjust α to α’ (0.05/3). Then, a probability value <0.0167 was considered to be statistically significant. One-way analysis of variance (ANOVA) was selected for comparison among multiple groups, and the least significant difference (LSD) or Tamhane’s T2 method were used for pairwise comparison among the groups. To further assess the prediction performance of the parameters in quantitative analysis, receiver operating characteristic (ROC) analysis was performed. SPSS 26.0 was utilized for all statistical analyses (SPSS Inc, Chicago, IL, USA). A probability value of less than 0.050 (*p* < 0.050) was considered significant.

## Results

### Clinical Characteristics of the Patients

**Figure 1:**
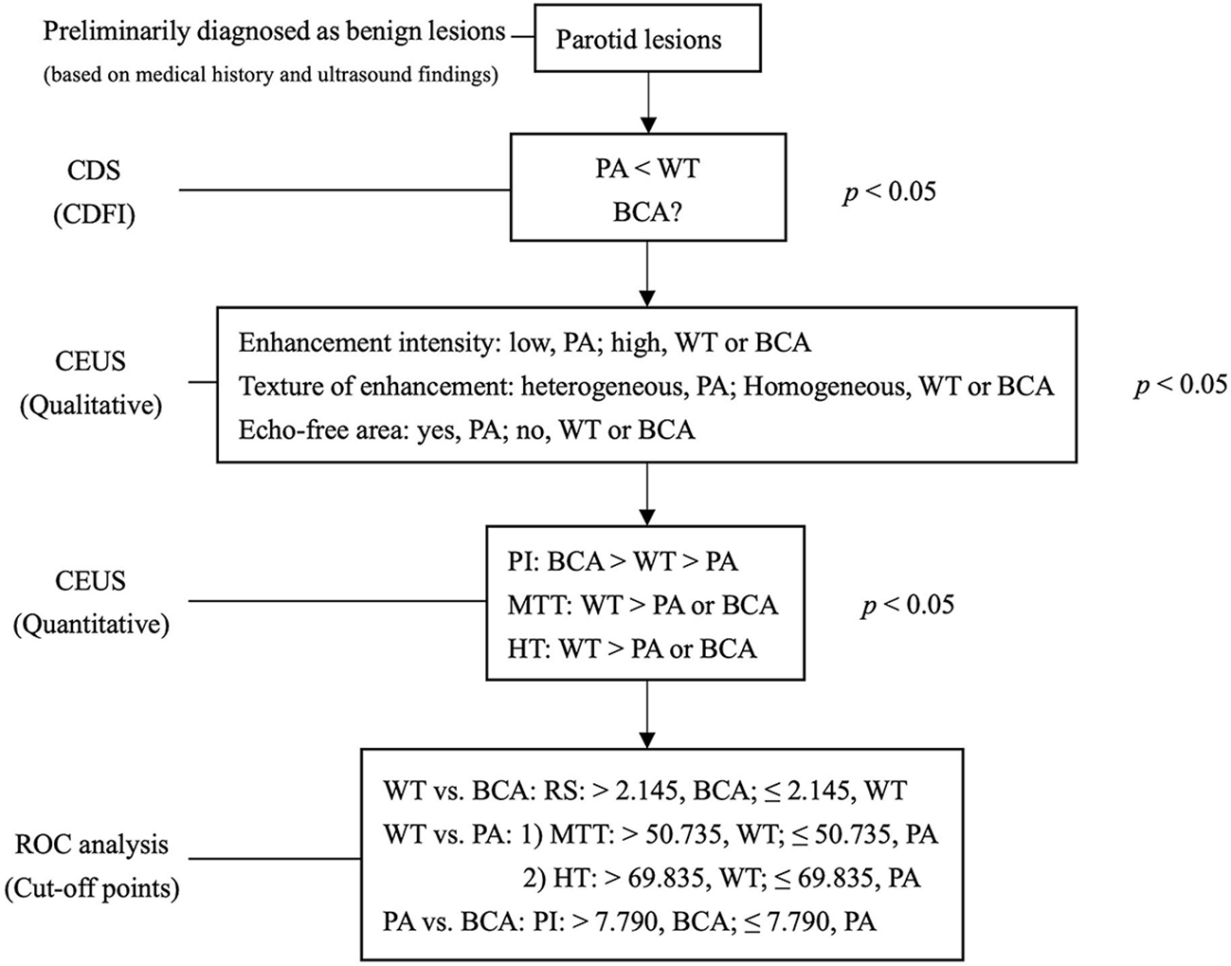
Multimodal diagnostic pathway. The salivary lesions were preliminarily diagnosed as benign lesions based on medical history and B-mode ultrasound. The grade of CDFI distinguishes hyper- from hypo-vascularised tumor entities initially. Qualitative analysis of CEUS showed specific features of lesions in the PA group and quantitative analysis of CEUS further differentiated the lesions in the BCA and WT groups. PA, pleomorphic adenoma; WT, Warthin tumor; BCA, basal cell adenoma; CEUS, contrast-enhanced ultrasound; CDFI, Color Doppler flow imaging; PI, peak intensity; MTT, mean transit time; HT, time from peak to one half; RS, rising slope of wash in curve; P < 0.050 was considered to be statistically significant. The optimal cutoff points calculated by ROC curve analysis are for diagnostic reference only.

The multimodal diagnostic pathway is depicted in **Figure 1**. The parotid lesions were preliminarily diagnosed as benign lesions based on the medical history and the B-mode US. A total of 105 patients (60 women and 45 men) with parotid gland tumors were included in this study. Among them, 32 patients were diagnosed as WT, 51 were established as PA, and 22 were found as BCA based on the clinicopathological results. The clinical characteristics of the patients belonging to the 3 groups are shown in **Table 1**. The differences in age and the number of lesions among the 3 benign groups were statistically significant (age: *p* < 0.001, Kruskal–Wallis test; the number of lesions: *p* = 0.005, Fisher’s exact test). Comparison between the 2 groups revealed that the mean values of patient age in the WT and BCA groups were significantly higher than those in the PA group (WT vs. PA: *p* < 0.001; BCA vs. PA: *p* < 0.001, Mann–Whitney U-test). When compared with the WT and PA groups in pairs, all patients in the BCA group had single masses (BCA vs. WT: *p* = 0.003, Fisher’s exact test; BCA vs. PA: *p* = 0.044, Fisher’s exact test).

**Table 1:** Detailed clinical and histopathological characteristics of patients with benign tumors in the parotid gland (n = 105).

Characteristics	WT	PA	BCA	*p*
**No. patients**	32 (30.5%)	51 (48.6%)	22 (20.9%)	
**Sex**				0.073
Female	13 (40.6%)	32 (62.7%)	15 (68.2%)	
Male	19 (59.4%)	19 (37.3%)	7 (31.8%)	
**Age**				**<0.001**
Mean	62	42^*^	62^#^	
Ranges	36 to 84	14 to 65^*^	48 to 74^#^	
**Number of lesions**				**0.005**
Single	22 (68.8%)	45 (88.2%)^*^	22 (100%)^*^	
Multiple	10 (31.2%)	6 (11.8%)^*^	0 (0%)^*^	

PA, pleomorphic adenoma; WT, Warthin tumor; BCA, basal cell adenoma.

P < 0.050 was considered statistically significant.

Compared with WT, ^*^p < 0.050 and compared with PA, ^#^p < 0.050.

According to Bonferroni's method, adjust α to α' (0.05/3). A probability value of less than 0.0167 was considered to be statistically significant. The bold values were considered to be statistically significant.

### Conventional US Findings

A total of 129 nodules (42 of WT, 65 of PA, and 22 of BCA) of benign parotid gland tumors were reviewed (**Table 2**). The differences among the 3 benign groups in terms of tumor size (*p* < 0.001, Kruskal–Wallis test) and blood-flow grade (*p* = 0.031, Fisher’s exact test) were statistically significant. The majority of WT (83.3%), PA (78.5%), and BCA (95.5%) in our study had a regular shape (oval or rounded). The margins were well-defined in all WT and BCA tumors and only 4.6% of the PA lesions showed a poor margin definition. Moreover, with regard to the echogenicity of tumors, all lesions were mainly solid echo, but WT (28.6%) and PA (24.6%) were slightly more mixed echo (solid and cystic) than BCA, which were almost all solid echo (95.5%). Considering the homogeneity of tumors, the majority of BCA (95.5%) lesions were homogenous, while WT (28.6%) and PA (24.6%) were more heterogeneous. When compared with the PA and BCA groups, the blood-flow grade was mainly 2–3 in the WT group (29/42, 69.0%), which means that WT had higher vascularity and the difference was statistically significant (*p* = 0.031, Fisher’s exact test) (**Figure 5**).

**Table 2:** Clinical and conventional US characteristics of nodules (n = 129) in the parotid gland.

Characteristics	WT (n = 42)	PA (n = 65)	BCA (n = 22)	*p*
**Diameter (mm)**				**<0.001**
Mean	31	24^*^	14^*#^	
Ranges	12 to 59	10 to 53^*^	8 to 23^*#^	
**Side (%)**				0.368
Left	25 (59.5%)	36 (55.4%)	16 (72.7%)	
Right	17 (40.5%)	29 (44.6%)	6 (27.3%)	
**Shape**				0.202
Regular (oval, rounded)	35 (83.3%)	51 (78.5%)	21 (95.5%)	
Irregular	7 (16.7%)	14 (21.5%)	1 (4.5%)	
**Margin definition**				0.287
Well defined	42 (100%)	62 (95.4%)	22 (100%)	
Poor defined	0 (0%)	3 (4.6%)	0 (0%)	
**Internal echo**				0.058
Solid	30 (71.4%)	49 (75.4%)	21 (95.5%)	
Mixed (Solid and cystic)	12 (28.6%)	16 (24.6%)	1 (4.5%)	
**Homogeneity**				0.058
Yes	30 (71.4%)	49 (75.4%)	21 (95.5%)	
No	12 (28.6%)	16 (24.6%)	1 (4.5%)	
**Blood flow grade of CDFI**				**0.031**
0–I	13 (31.0%)	37 (56.9%) ^*^	10 (45.5%)	
II–III	29 (69.0%)	28 (43.1%) ^*^	12 (54.5%)	

PA, pleomorphic adenoma; WT, Warthin tumor; BCA, basal cell adenoma; CDFI, color Doppler flow imaging. Diameter represents the largest diameter of the tumors from the US reports. P < 0.050 was considered to be statistically significant.

Compared with WT, ^*^p < 0.05 and compared with PA, ^#^p < 0.05.

According to Bonferroni's method, adjust α to α' (0.05/3). A probability value of less than 0.0167 was considered to be statistically significant. The bold values were considered to be statistically significant.

### CEUS Findings

#### Qualitative Analysis of CEUS

**Figure 2:**
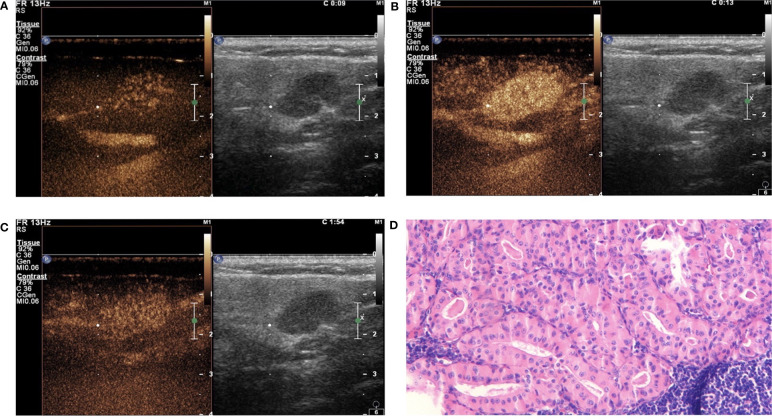
Warthin tumor (WT) in contrast-enhanced ultrasound (CEUS). The qualitative CEUS indicated the mass with highly intensive and homogeneous enhancement and the perfusion pattern of contrast agent was “fast in and slow out” (**A**-early phase; **B**-middle phase; **C**-late phase). **(D)** H&E stain (original magnification ×100).

**Figure 3:**
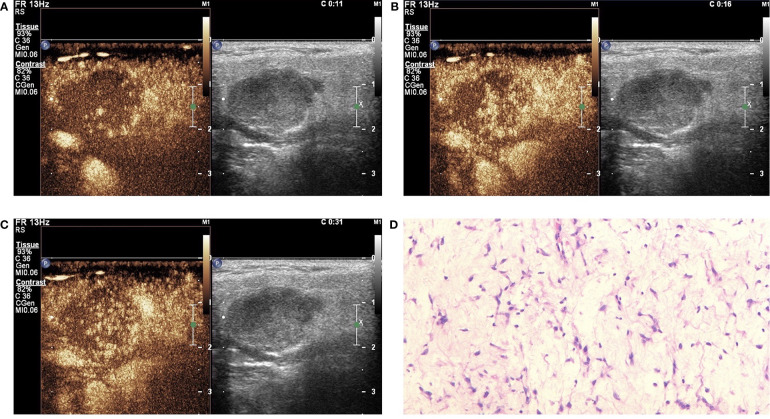
Pleomorphic adenoma (PA) in contrast-enhanced ultrasound (CEUS). The qualitative CEUS showing the mass with low intensity and heterogeneous enhancement and echo-free areas. The perfusion pattern of contrast agent was “slow in and fast out” (**A**-early phase; **B**-middle phase; **C**-late phase). **(D)** H&E stain (original magnification ×100).

**Figure 4:**
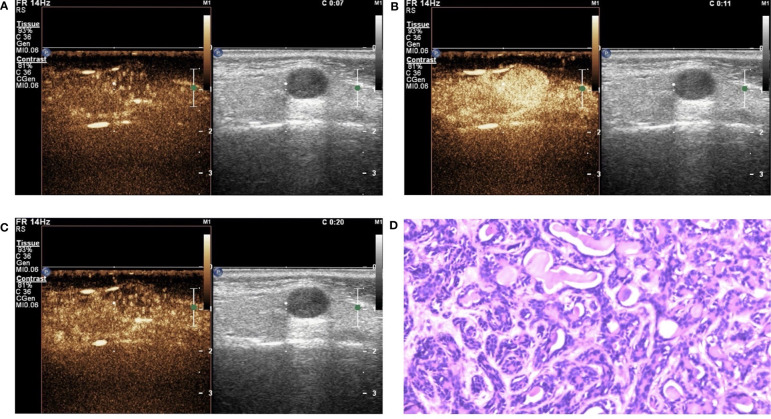
Basal cell adenoma (BCA) in contrast-enhanced ultrasound (CEUS). The qualitative CEUS showing the mass with high intensity and homogeneous enhancement. The perfusion pattern of contrast agent was “fast in and fast out” (**A**-early phase; **B**-middle phase; **C**-late phase). **(D)** H&E stain (original magnification ×100).

In this study, the 110 cases of parotid gland masses were divided into 3 groups: WT (34 cases), PA (54 cases), and BCA (22 cases) (**Table 3**) (**Figures 2**–**4**). The following findings were summarized by observing from the perfusion kinetics of CEUS: Margin after enhancement were mostly clear and features such as ring enhancement and larger size after enhancement was rarely noted in all lesions. The majority of CEUS images of the WT lesions were highly enhanced (91.2%) (when compared with the surrounding normal parotid gland tissues) and homogeneous enhancement (70.6%). Among all the WT lesions, 25 lesions (25/34, 73.5%) were “fast in” and 30 lesions were (30/34, 88.2%) “slow out”. All the BCA lesions were high enhancement (100%) and mostly homogeneous enhancement (81.8%), with a “fast in” (20/22, 90.9%) and “fast out” (18/22, 81.8%) pattern. However, most of PA lesions showed low enhancement (88.9%) and had a heterogeneous texture of enhancement (94.4%), presenting with an echo-free area (96.2%) and a pattern of “slow in” (46/54, 85.2%) and “fast out” (44/54, 81.5%). When paired comparisons were made in pairs, the lesions in the PA group (48, 88.9%) were of lower enhancement (when compared with the surrounding normal tissues) than those in the WT and BCA groups (PA vs. WT: *p* < 0.001, χ^2^ test; PA vs. BCA: *p* < 0.001, Fisher’s exact test). When compared with the WT and BCA groups, the lesions in the PA group were more heterogeneous in enhancement (PA vs. WT: *p* < 0.001, χ^2^ test; PA vs. BCA: *p* < 0.001, χ^2^ test). The echo-free areas in the lesions were more observed in the PA group than in the other two groups, and the difference was statistically significant (PA vs. WT: *p* < 0.001, χ^2^ test; PA vs. BCA: *p* < 0.001, Fisher’s exact test). In addition, the wash-in pattern of the SonoVue sign was from the border of the mass into the center concentration to the peak-value slowly (“slow in”) in lesions in the PA group than those in the WT and BCA groups (*p* < 0.001, χ^2^ test). With regard to the wash-out pattern of the SonoVue sign, the signal intensity reduced gradually after remaining stable for some time (“slow out”) in lesions in the WT group than those in the PA and BCA groups (*p* < 0.001, χ^2^ test).

**Table 3:** The real-time dynamic results and qualitative analysis of contrast-enhanced ultrasonography (CEUS) in common benign parotid gland tumors (n = 110).

Qualitative variables	WT (n = 34)	PA (n = 54)	BCA (n = 22)	*p*
**Enhancement intensity**				**<0.001**
High	31 (91.2%)	6 (11.1%)^*^	22 (100%)^#^	
Low	3 (8.8%)	48 (88.9%)^*^	0 (0%)^#^	
**Texture of enhancement**				**<0.001**
Homogeneous	25 (73.5%)	3 (5.6%)^*^	18 (81.8%)^#^	
Heterogeneous	9 (26.5%)	51 (94.4%)^*^	4 (18.2%)^#^	
**Margin after enhancement**				1.000
Clear	33 (97.1%)	52 (96.2%)	21 (95.4%)	
Unclear	1 (2.9%)	2 (3.8%)	1 (4.6%)	
**Ring enhancement**				0.301
Yes	0 (0%)	3 (5.6%)	0 (0%)	
No	34 (100%)	51 (94.4%)	22 (100%)	
**Echo-free area**				**<0.001**
Yes	3 (8.8%)	52 (96.2%)^*^	0 (0%)^#^	
No	31 (91.2%)	2 (3.8%)^*^	22 (100%)^#^	
**Size of the enhanced lesions**				1.000
Larger	1 (2.9%)	3 (5.6%)	1 (4.5%)	
Similar	33 (97.1%)	51 (94.4%)	21 (95.5%)	
**Wash-in pattern**				**<0.001**
Fast in	25 (73.5%)	8 (14.8%)^*^	20 (90.9%)^#^	
Slow in	9 (26.5%)	46 (85.2%)^*^	2 (9.1%)^#^	
**Wash-out pattern**				**<0.001**
Fast out	4 (11.8%)	44 (81.5%)^*^	18 (81.8%)^*^	
Slow out	30 (88.2%)	10 (18.5%)^*^	4 (18.2%)^*^	

PA, pleomorphic adenoma; WT, Warthin tumor; BCA, basal cell adenoma.

P < 0.050 was considered to be statistically significant; CEUS, contrast-enhanced ultrasound.

Compared with WT, ^*^p < 0.050 and compared with PA, ^#^p < 0.050. The bold values were considered to be statistically significant.

#### Quantitative Analysis of CEUS

**Figure 5:**
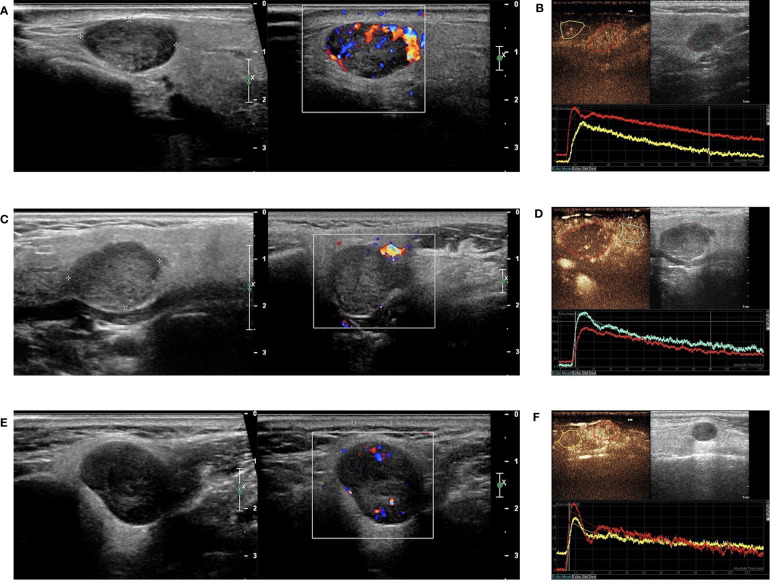
B-mode sonography, Color Doppler sonography (CDs), and dual-imaging of CEUS and gray-scale US with ROI in the remarkable perfusion areas in both the lesions and the surrounding tissues. **(A)** Hypoechoic and regular masses with well-defined border in the parotid glands of patients with WT, and CDs displayed marked intratumal vascularity in the lesion (grade III); **(B)** TIC analysis by the QLAB software showed that the red line represented the lesion of WT, while the yellow line represented the surrounding tissues; **(C)** Hypoechoic and regular masses with a well-defined border in the parotid glands of patients with PA and CDs showing no blood flow signal in the lesion (grade 0); **(D)** TIC analysis by the QLAB software showing that the red line represented the lesion of PA, while the green line represented the surrounding tissues; **(E)** hypoechoic and regular masses with a well-defined border in the parotid glands of patients with BCA and CDs showing a small amount of blood flow in the lesion (grade II); **(F)** TIC analysis by the QLAB software showing that the red line represented the lesion of BCA, while the yellow line represents the surrounding tissues. ROI, region of interest; TIC, time-intensity curve.

**Figure 6:**
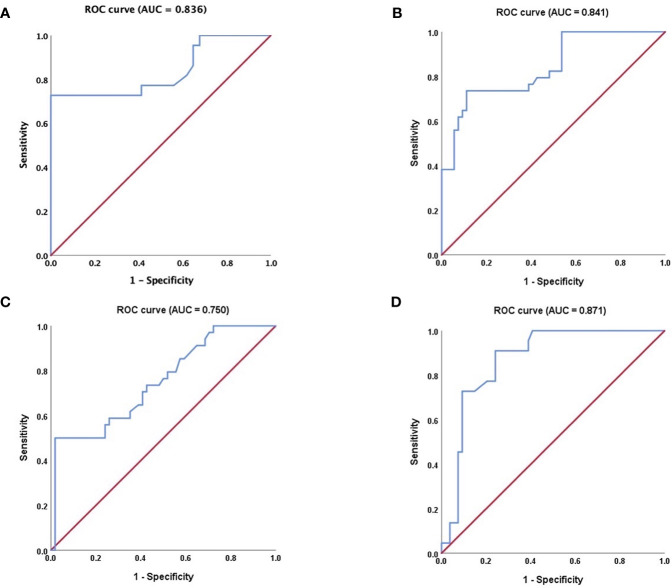
Receiver operating characteristic (ROC) curve analysis. **(A)** regarding RS, ROC curve for WT and BCA tumors (P < 0.001, AUC = 0.836); **(B)** regarding MTT, ROC curve for WT and PA tumors (P < 0.001 and AUC = 0.841); **(C)** regarding HT, ROC curve for WT and PA tumors (P < 0.001 and AUC = 0.750, respectively); **(D)** regarding PI; ROC curve for PA and BCA tumors (P < 0.001 and AUC = 0.871). ROC, receiver operating characteristic; AUC, area under the curve; RS, rising slope; MTT, mean transit time; HT, time from peak to one half, PI, peak intensity. The nonparametric estimate of the AUC and its 95% confidence interval are shown respectively. The non-parametric estimate of AUC is the sum of the areas of the trapezoids formed by connecting the points on the ROC curve. Please refer to the **Supplementary Materials** for details.

The results of the quantitative parameters of the TICs are summarized in **Table 4** (**Figure 5**). No significant differences were noted in RT, TTP, and RS; however, PI, MTT, AUC, and HT exhibited significant differences (PI: *p* < 0.001, MTT: *p* < 0.001, AUC: *p* < 0.001, and HT: *p* < 0.001, one-way ANOVA) (**Table 4**). To be specific, when compared in pairs, the mean values of the PI in the BCA group were significantly higher than those in the WT and PA groups (BCA vs. WT: *p* = 0.001; BCA vs. PA: *p* < 0.001, LSD method). The mean values of MTT (WT vs. PA: *p* < 0.001; WT vs. BCA: *p* < 0.001, Tamhane’s T2 method) and HT (WT vs. PA: *p* < 0.001; WT vs. BCA: *p* < 0.001, Tamhane’s T2 method) in the WT group were significantly higher when compared with those in the PA and BCA groups. The mean values of AUC in the WT and BCA groups were higher than those in the PA group (WT vs. PA: *p* = 0.009; BCA vs. PA: *p* < 0.001, Tamhane’s T2 method). The optimal cutoff points for the parameters of quantitative analysis were calculated (**Figures 1** and **6**). ROC analysis was applied to determine these points. With regard to RS, the optimal cutoff point was 2.145, which was identified to distinguish between the lesions in the WT and BCA groups (*p* < 0.001 and AUC = 0.836, ROC analysis). In the case of MTT and HT, the optimal cutoff points were 50.735 and 69.835, respectively, which were discerned to distinguish between the lesions in the WT and PA groups (MTT: *p* < 0.001 and AUC = 0.841; HT: *p* < 0.001 and AUC = 0.750, respectively, ROC analysis). In addition, regarding PI, the optimal cutoff point was 7.790, which was determined to distinguish between the lesions in the PA and BCA groups (*p* < 0.001 and AUC = 0.871).

**Table 4:** Comparison of quantitative parameters of time-intensity curves (TICs) by contrast-enhanced ultrasonography (CEUS) for common benign parotid gland tumors (Mean ± SD) (n = 110).

Group	n	RT (s)	PI (dB)	MTT (s)	AUC	HT (s)	TTP (s)	RS (dB/s)
**WT**	34	4.36 ± 1.87	7.45 ± 1.38	63.90 ± 23.90	536.27 ± 192.76	66.08 ± 22.18	16.89 ± 2.78	1.28 ± 0.44
**PA**	54	4.08 ± 1.52	6.74 ± 1.80^*^	36.75 ± 11.82^*^	418.70 ± 137.40^*^	46.29 ± 14.85^*^	16.23 ± 6.08	2.42 ± 3.34
**BCA**	22	3.73 ± 1.59	8.93 ±1.13^*#^	40.55 ± 7.57^*^	552.15 ± 82.33^#^	45.93 ± 14.50^*^	15.49 ± 3.98	2.61 ± 0.99
**F**		0.99	15.30	31.51	9.59	15.38	0.56	2.92
***p***		0.376	**<0.001**	**<0.001**	**<0.001**	**<0.001**	0.572	0.058

PA, pleomorphic adenoma; WT, Warthin tumor; BCA, basal cell adenoma; CEUS, contrast-enhanced ultrasound; RT, rise time; PI, peak intensity; MTT, mean transit time; AUC, area under curve; HT, time from peak to one half; TTP, time to peak; RS, rising slope of wash in curve; SD, standard deviation. Data are shown as the mean ± SD. P < 0.050 was considered to be statistically significant.

Compared with WT, ^*^p < 0.050 and compared with PA, ^#^p < 0.050.

Please refer to the **Supplementary Materials** for details. The bold values were considered to be statistically significant.

## Discussion

The parotid gland is the most common location for the onset of salivary tumors. It is obvious from the literature that PA and WT are the most common benign lesions of the parotid gland, which account for 83–93% of benign parotid glands. The next most common tumor is BCA. On the contrary, malignant lesions are relatively rare and usually include different tissue cell types in the parotid gland (25, 26). For benign tumors in the parotid gland, distinguishing PA allows planning of the correct timing of surgery, considering that this histological type has the potential for malignant transformation in 5.0–9.8% of all cases. Delays in the operation time may increase the risk of malignant transformation. Therefore, the operation should be performed as soon as possible (4, 27). Moreover, among the benign tumors of the salivary gland, PA has a recurrence rate of 6.8%. It has been shown that the most important reason for recurrence of PA is enucleation with rupture and incomplete tumor resection in surgery (28). Thus, ideally, PA should be diagnosed before surgery to facilitate the selection of an appropriate surgical approach. Histologically, BCA has 4 morphological subtypes: trabecular, tubular, solid, and membranous, of which the unique membranous subtype is common in men and characterized by the highest risk (up to 28%) of malignant transformation owing to the lack of capsule (29). The diagnosis of BCA by FNAB is challenged due to the overlapping features with adenoid cystic carcinoma, basal cell adenocarcinoma, and PA (30). Therefore, it is best to perform a preliminary diagnosis before surgery. In addition, most studies on BCA have only been case reports, and only a few studies have summarized the available data. As for WT, the malignant transformation rate is extremely rare in 0.3% of all cases (31). Therefore, unlike PA and BCA, a relatively mild elective surgery was selected. Considering the superficial location of the tumor, less invasive surgical approaches such as superficial parotidectomy (avoid rupture of the tumor capsule) or local excision with the surrounding tissue can be applied. The local recurrence rate of WT is low. Once recurrence occurs, possibly owing to multifocal tumors or insufficient resection (31). In our study, WT showed a high tendency of multiple lesions (10/32, 31.2%), which is consistent with other research results (32, 33). Furthermore, we found that the majority of the patients with WT (19, 59.4%) were male. Although there are some recent reports indicating that the difference in gender ratio is decreasing and the occurrence of WT remains male preference (34). In other words, when a second tumor appears, it should be asked whether “is this a real recurrence or is it a tumor left over from the first surgical exploration?” Multifocality and bilaterality are of great importance to heavy smokers, therefore preoperative imaging examinations should be performed thoroughly to allow these patients to benefit by quitting smoking (7).

With the advancement in technology in recent years, the diagnosis of salivary gland lesions has also improved. B–mode US is a cost-effective tool for the detection of salivary gland lesions. Ideally, it is recommended to not only distinguish benign and malignant conditions of a lesion before surgery but also to conduct a preliminary diagnosis of the pathological type tendency. Although several types of researches have recently attempted to distinguish between benign and malignant tumors of the parotid gland by various US parameters (10, 25), a precise classification system is lacking. In our study, the majority of WT, PA, and BCA had regular shapes, with well-defined margins by the US. It is not enough to distinguish these three diseases only by the conventional ultrasound. However, color Doppler ultrasonography revealed the difference in vascularity between WT and PA in our study, although no difference was noted between PA and BCA or WT and BCA. The degree of vascularity in WT was high, which was consistent with the reports of some previous studies in which tumors of WT were reported to have a rich blood supply (4, 35). Nevertheless, CDS alone cannot be used as a dependable assessment considering that it relies mainly on personal clinical expertise rather than on measurable evidence and that different brands of machines vary in their sensitivities for blood flow imaging. Taking head and neck in the US as an example, recent studies have indicated that the application of CDS and CEUS is beneficial. However, the predictive value of a single technology remains extremely low (36). Thus, more precise imaging diagnostic methods should be essentially applied before operation.

In order to obtain a more accurate diagnosis, we attempted to apply CEUS to describe the micro-vascularity of lesions. The exploration of the diagnosis of solid salivary tumors by the US requires a multimodal approach. It has been reported that CEUS can distinguish between benign and malignant tumors. When assessed by CEUS, the strong diagnostic criteria for malignant entities include the unclear margin of the glands, uneven angiogenesis, and non-uniform structure or distribution of the circulating beds. The diagnostic criteria for benign tumors include clear margin, mild inhomogeneous structure, homogeneous vascularity, or distribution of circulating beds (37). We explored the application of CEUS to distinguish among the common benign parotid gland tumors. PA lesions were mostly found to have low enhancement (88.9%) and heterogeneous texture of enhancement (94.4%), with the presentation of echo-free area (96.2%) and a pattern of “slow in” (46/54, 85.2%) and “fast out” (44/54, 81.5%) in this study. These observations may be attributed to PA evolving from a benign glandular epithelial tumor, which grows slowly with a sparse vascular distribution (38). This was presumed to be the reason for the low enhancement pattern of PA. Furthermore, the chief reason for the heterogeneous enhancement of the PA lesions is that the tissue contains a large amount of myxoid and necrotic components. Myxoid components are not characteristically anechoic in the two-dimensional US; hence, this technique alone cannot be used to distinguish the myxoid components of the PA lesions in clinical practice. Therefore, CEUS is needed for further identification. A study of 100 cases by Stennert et al. established that the myxoid (stroma-rich) subtype accounted for 71.0% of the PAs. The pathological features of the myxoid type in PAs of the parotid gland were abundant myxoid ground-substances with interspersed spindle and stellate cells, but without closely related epithelial islands (39). PAs are usually described as polycyclic or lobular tumors. Histologically, they are composed of epithelial and myoepithelial cells. PAs may exhibit cystic changes and hemorrhage in the tumors, especially when they are large (8). In our study, PA could be initially distinguished by using the simple diagnostic method of qualitative CEUS. The common features of CEUS images of WT and BCA lesions were high (WT: 91.2%, BCA: 100%) and relatively homogeneous (WT: 70.6%, BCA: 81.8%) enhancement in our study. WTs are derived from the lymphoid tissues and therefore exhibit a spreading and high enhancement, similar to that in inflammatory lymph nodes. Woo et al. (40) reported that WTs have a densely packed, capillary-like network of vessels with high vascularity, albeit for those with a higher cystic component. In the latter cases, the perfusion is more “septal-like”, with no such pronounced perfusion pattern, making diagnosis more difficult. It has been reported that these cystic areas may represent the histopathological patterns of Warthin’s tumors. “The epithelial layer is arranged in branching, cystic or fissured space”, which may be related to the cystic degeneration or focal necrosis (41). In order to avoid the interference of the large cystic area on the diagnosis, we attempted to avoid the large cystic area when selecting the ROI, but we mainly analyzed the substantive portion of the lesions. It has been documented that similar to WT, BCA has several vascular channels in the endothelial lining, with obvious small capillaries and venules (42). This may be the reason why WT and BCA were highly enhanced in our current study and, we speculated that, except from WT and BCA, other types of MA benign tumors of salivary gland may also share the similar characteristics. It is difficult to distinguish between WT and BCA only by qualitative CEUS imaging. We also found that the perfusion pattern of the contrast agent in the WT lesions were mostly “slow out” when compared with the other 2 groups (*p* < 0.001, χ^2^ test), and the perfusion pattern of the contrast agent in the PA lesions were mostly “slow in” when compared with the other 2 groups (*p* < 0.001, χ^2^ test). In order to quantify our observations, we used a quantitative analysis of CEUS. What is the reason for the “slow in” pattern observed in our study? It might also be explained that these benign mixed tumors of PA are arranged with different morphological patterns and subtypes, with an uneven and tortuous distribution of the blood vessels (43). We applied CEUS that even describes the micro-vascularity of the lesions with echo-free areas and provides measurable perfusion kinetics. Perfusion and its kinetics were also investigated for MRI. The features of PA by MRI have been reported in the literature as a well-defined mass and the solid portion of PA was noted with hyperintensity on T2-weighted imaging (T2WI), reflecting the myxoid and/or chondroid stromal components of PA. The solid portion of the PA usually demonstrates a persistent pattern on dynamic contrast-enhanced MRI (44). WT is often depicted as a well-defined mass in MRI, typically consisting of different ratios of solid and cystic components, with the solid portion found to have an intensity to hypointensity on T2WI. A washout pattern on dynamic contrast-enhanced MRI has been reported in the literature (45). The MRI revealed that BCA is a clear oval-shaped lesion. When compared with the parotid gland parenchyma of T2WI, the solid content of BCA demonstrated homogeneous isointensity to hypointensity (46). However, Takita et al. noted no marked differences in CT or MRI features between the PA and BCA lesions (47). For the most common tumors of the parotid gland, such as PA and WT, in our study, we noted that qualitative CEUS enables PA to be clearly diagnosed first. Then, we aimed at distinguishing WT from BCA. Takita et al. (47) claimed that it is crucial to distinguish BCA from low-grade malignancies, especially in patients who showed an increase in the tumor size, as well as some signs of malignancy or an intermediate pattern on the US or CT. However, past studies have reported that the accuracy rate of FNAB in diagnosing BCA is significantly lower than that of PA. Thus, the parameters of TIC analysis by quantitative analyses of CEUS were considered in this study.

The main goal of quantitative analysis of CEUS is to further distinguish BCA and WT and to evaluate parameters that may be used for identification among different groups of the 3 common tumors in the parotid gland. In the analysis of the quantitative parameters, when compared in pairs, the mean values of the PI in the BCA group were significantly higher than those in the WT and PA groups (BCA vs. WT: *p* = 0.001; BCA vs. PA: *p* < 0.001, LSD method), which is in agreement with the previous result that BCA is a hyper-vascularized tumor. The differences among the data (PI, MTT, AUC, and HT) of the 3 groups were statistically significant (PI: *p* < 0.001, MTT: *p* < 0.001, AUC: *p* < 0.001, and HT: *p* < 0.001, one-way ANOVA). ROC analysis was applied to determine the optimal cutoff points. Regarding RS, the point was 2.145, which was identified to distinguish between the WT and BCA lesions (*p* < 0.001 and AUC = 0.836) and may be used as a predictive model. In short, when the parotid gland lesions are initially judged to be benign by clinicians and multiple diagnostic tools (such as the US, CDS, and qualitative CEUS) are applied, PA usually can be distinguished first, and then these optimal cutoff points of different quantitative CEUS parameters might be used for differentiation between WT and BCA. Especially, when RS was >2.145, the mass was more likely to be diagnosed as BCA, and the other optimal cut-off points of MTT, HT, and PI may provide clues to the diagnosis of the common 3 benign tumors in the parotid gland. It is crucial to establish the type of the parotid gland tumor before surgery to determine the timing of surgery and to select the appropriate therapeutic modality. Preoperative histological examination of parotid tumors is of great significance in determining the extent of resection and for preserving the facial nerve. Considering the low incidence rate of benign tumors in other pathological types, except PA, WT, and BCA, and our limited research samples, further in-depth differential diagnosis is difficult. A large sample, multicenter, and prospective study is warranted in the future. The comprehensive analysis of US, CDS, and qualitative and quantitative CEUS in our study may provide additional data and novel ideas for radiologists to precisely identify the 3 major benign lesions of the parotid glands.

## Conclusions

The special image characteristics of qualitative CEUS might serve as a diagnostic criterion for PA in the parotid gland. However, for MA including WT and BCA, there is no obvious distinguishing specificity of characteristics in qualitative CEUS. The parameters of quantitative analysis of CEUS may provide valuable diagnostic information. The optimal cut-off points may provide a novel method to further differentiate in pairs among groups of the 3 common benign tumors in the parotid gland, especially for distinguishing WT from BCA. The identification of these 3 diseases before surgery may be of great significance for the selection of operation time and surgical methods and changes in the patient’s lifestyle.

## Data Availability Statement

The raw data supporting the conclusions of this article will be made available by the authors, without undue reservation.

## Ethics Statement

Written informed consent was obtained from the individual(s), and minor(s)’ legal guardian/next of kin, for the publication of any potentially identifiable images or data included in this article.

## Author Contributions

MY conducted the experimental progress and manuscript writing. DX and LC performed the manuscript revision. LZ was responsible for the experimental design. All authors contributed to the article and approved the submitted version.

## Conflict of Interest

The authors declare that the research was conducted in the absence of any commercial or financial relationships that could be construed as a potential conflict of interest.

## Abbreviations

CEUS, contrast-enhanced ultrasound; PA, pleomorphic adenomas; WT, Warthin tumor; BCA, basal cell adenoma; CDs, color Doppler sonography; TIC, time-intensity curve; PI, peak intensity; RT, rise time; MTT, mean transit time; AUC, area under curve; HT, time from peak to one half; TTP, time to peak; RS, rising slope of wash in the curve; SD, standard deviation; ROC, receiver operating characteristic; CDFI, color Doppler flow imaging; DWI, diffusion-weighed imaging; MA, monomorphic adenoma.

## References

[1] Dostalova L Kalfert D Jechova A Koucky V Novak S Kuchar M. The Role of Fine-Needle Aspiration Biopsy (FNAB) In the Diagnostic Management of Parotid Gland Masses With Emphasis On Potential Pitfalls. Eur Arch Otorhinolaryngol (2020) 277(6):1763–69. 10.1007/s00405-020-05868-1 32107613

[2] Fujita Y Yoshida T Sakakura Y Sakakura T. Reconstruction of Pleomorphic Adenoma of The Salivary Glands In Three-Dimensional Collagen Gel Matrix Culture. Virchows Arch (1999) 434(2):137–43. 10.1007/s004280050317 10071248

[3] Zhan KY Khaja SF Flack AB Day TA. Benign Parotid Tumors. Otolaryngol Clin North Am (2016) 49(2):327–42. 10.1016/j.otc.2015.10.005 27040584

[4] Rzepakowska A Osuch-Wójcikiewicz E Sobol M Cruz R Sielska-Badurek E Niemczyk K. The Differential Diagnosis of Parotid Gland Tumors With High-Resolution Ultrasound In Otolaryngological Practice. Eur Arch Otorhinolaryngol (2017) 274(8):3231–40. 10.1007/s00405-017-4636-2 PMC5500678 28612315

[5] Chen KT. Carcinoma Arising In Monomorphic Adenoma of the Salivary Gland. Am J Otolaryngol (1985) 6(1):39–41. 10.1016/s0196-0709(85)80007-7 3977010

[6] Nagao T Gnepp DR Rhw S Vielh P. Warthin tumour. In: El-Naggar K Chan JK Grandis JR Takata T Slootweg PJ, editors. WHO Classification of Head and Neck Tumors, 4th. Lyon: IARC Press (2017a).

[7] Hellquist H Paiva-Correia A Vander Poorten V Quer M Hernandez-Prera JC Andreasen S. Analysis of the Clinical Relevance of Histological Classification of Benign Epithelial Salivary Gland Tumours. Adv Ther (2019) 36(8):1950–74. 10.1007/s12325-019-01007-3 PMC6822986 31209701

[8] Lee YY Wong KT King AD Ahuja AT. Imaging of Salivary Gland Tumours. Eur J Radiol (2008) 66(3):419–36. 10.1016/j.ejrad.2008.01.027 18337041

[9] Białek EJ Jakubowski W Karpińska G. Role of Ultrasonography In Diagnosis and Differentiation of Pleomorphic Adenomas: Work In Progress. Arch Otolaryngol Head Neck Surg (2003) 129(9):929–33. 10.1001/archotol.129.9.929 12975263

[10] Bozzato A Zenk J Greess H Hornung J Gottwald F Rabe C. Potential of Ultrasound Diagnosis for Parotid Tumors: Analysis of Qualitative And Quantitative Parameters. Otolaryngol Head Neck Surg (2007) 137(4):642–6. 10.1016/j.otohns.2007.05.062 17903584

[11] Alyas F Lewis K Williams M Moody AB Wong KT Ahuja AT. Diseases of the Submandibular Gland as Demonstrated Using High Resolution Ultrasound. Br J Radiol (2005) 78(928):362–9. 10.1259/bjr/93120352 15774602

[12] Sodhi KS Bartlett M Prabhu NK. Role of High Resolution Ultrasound In Parotid Lesions In Children. Int J Pediatr Otorhinolaryngol (2011) 75(11):1353–8. 10.1016/j.ijporl.2011.07.005 21816492

[13] Salgarelli AC Cappare P Bellini P Collini M. Usefulness of Fine-Needle Aspiration In Parotid Diagnostics. Oral Maxillofac Surg (2009) 13(4):185–90. 10.1007/s10006-009-0182-4 19821124

[14] Pratap R Qayyum A Ahmed N Jani P Berman LH. Ultrasound-Guided Core Needle Biopsy of Parotid Gland Swellings. J Laryngol Otol (2009) 123(4):449–52. 10.1017/s0022215108003563 18826660

[15] Howlett DC. Diagnosing A Parotid Lump: Fine Needle Aspiration Cytology or Core Biopsy? Br J Radiol (2006) 79(940):295–7. 10.1259/bjr/74329476 16585720

[16] Nagao T L L T L P V MD W. Salivary Duct Carcinoma. In: El-Naggar K Chan JK Grandis JR Takata T Slootweg PJ, editors. WHO Classification of Head and Neck Tumors, 4th. Lyon: IARC Press (2017b).

[17] Quaia E. Microbubble Ultrasound Contrast Agents: An Update. Eur Radiol (2007) 17(8):1995–2008. 10.1007/s00330-007-0623-0 17351779

[18] Wilson SR Burns PN. Microbubble-Enhanced US In Body Imaging: What Role? Radiology (2010) 257(1):24–39. 10.1148/radiol.10091210 20851938

[19] Marotti J Heger S Tinschert J Tortamano P Chuembou F Radermacher K. Recent Advances of Ultrasound Imaging In Dentistry–A Review of the Literature. Oral Surg Oral Med Oral Pathol Oral Radiol (2013) 115(6):819–32. 10.1016/j.oooo.2013.03.012 23706922

[20] Greis C. Ultrasound Contrast Agents as Markers of Vascularity and Microcirculation. Clin Hemorheol Microcirc (2009) 43(1-2):1–9. 10.3233/ch-2009-1216 19713597

[21] Greis C. Quantitative Evaluation of Microvascular Blood Flow By Contrast-Enhanced Ultrasound (CEUS). Clin Hemorheol Microcirc (2011) 49(1-4):137–49. 10.3233/ch-2011-1464 22214685

[22] Gou JM Chen Q Zhou Q Liu YX. Quantitative Diagnosis of Salivary Gland Tumors With Contrast-Enhanced Ultrasound–A Preliminary Study. Oral Surg Oral Med Oral Pathol Oral Radiol (2013) 116(6):784–90. 10.1016/j.oooo.2013.09.013 24209995

[23] Strieth S Siedek V Rytvina M Gürkov R Berghaus A Clevert DA. Dynamic Contrast-Enhanced Ultrasound for Differential Diagnosis of Submandibular Gland Disease. Eur Arch Otorhinolaryngol (2014) 271(1):163–9. 10.1007/s00405-013-2523-z 23625388

[24] Adler DD Carson PL Rubin JM Quinn-Reid D. Doppler Ultrasound Color Flow Imaging in the Study of Breast Cancer: Preliminary Findings. Ultrasound Med Biol (1990) 16(6):553–9. 10.1016/0301-5629(90)90020-d 2238263

[25] Knopf A Mansour N Chaker A Bas M Stock K. Multimodal Ultrasonographic Characterisation of Parotid Gland Lesions–A Pilot Study. Eur J Radiol (2012) 81(11):3300–5. 10.1016/j.ejrad.2012.01.004 22269165

[26] Bradley PJ McGurk M. Incidence of Salivary Gland Neoplasms in A Defined UK Population. Br J Oral Maxillofac Surg (2013) 51(5):399–403. 10.1016/j.bjoms.2012.10.002 23103239

[27] Lewis AG Tong T Maghami E. Diagnosis and Management of Malignant Salivary Gland Tumors of the Parotid Gland. Otolaryngol Clin North Am (2016) 49(2):343–80. 10.1016/j.otc.2015.11.001 27040585

[28] Witt RL Eisele DW Morton RP Nicolai P Poorten VV Zbären P. Etiology and Management of Recurrent Parotid Pleomorphic Adenoma. Laryngoscope (2015) 125(4):888–93. 10.1002/lary.24964 25289881

[29] Yu GY Ubmüller J Donath K. Membranous Basal Cell Adenoma of the Salivary Gland: A Clinicopathologic Study of 12 Cases. Acta Otolaryngol (1998) 118(4):588–93. 10.1080/00016489850154775 9726688

[30] Kawahara A Harada H Akiba J Yokoyama T Kage M. Fine-Needle Aspiration Cytology of Basal Cell Adenoma of the Parotid Gland: Characteristic Cytological Features And Diagnostic Pitfalls. Diagn Cytopathol (2007) 35(2):85–90. 10.1002/dc.20598 17230571

[31] Therkildsen MH Christensen N Andersen LJ Larsen S Katholm M. Malignant Warthin’s Tumour: A Case Study. Histopathology (1992) 21(2):167–71. 10.1111/j.1365-2559.1992.tb00366.x 1505934

[32] Maiorano E Lo Muzio L Favia G Piattelli A. Warthin’s Tumour: A Study Of 78 Cases With Emphasis on Bilaterality, Multifocality and Association With Other Malignancies. Oral Oncol (2002) 38(1):35–40. 10.1016/s1368-8375(01)00019-7 11755819

[33] Zeebregts CJ Mastboom WJ van Noort G van Det RJ. Synchronous Tumours of the Unilateral Parotid Gland: Rare or Undetected? J Craniomaxillofac Surg (2003) 31(1):62–6. 10.1016/s1010-5182(02)00165-8 12553929

[34] A RR Rehani S Bishen KA Sagari S. Warthin’s Tumour: A Case Report and Review on Pathogenesis and its Histological Subtypes. J Clin Diagn Res (2014) 8(9):Zd37–40. 10.7860/jcdr/2014/8503.4908 PMC4225997 25386545

[35] Wei X Li Y Zhang S Li X Wang H Yong X. Evaluation Of Microvascularization in Focal Salivary Gland Lesions by Contrast-Enhanced Ultrasonography (CEUS) and Color Doppler Sonography. Clin Hemorheol Microcirc (2013) 54(3):259–71. 10.3233/ch-131732 23648453

[36] Bhatia KS Rasalkar DD Lee YP Wong KT King AD Yuen HY. Evaluation of Real-Time Qualitative Sonoelastography of Focal Lesions in the Parotid and Submandibular Glands: Applications and Limitations. Eur Radiol (2010) 20(8):1958–64. 10.1007/s00330-010-1756-0 20407904

[37] Badea AF Bran S Tamas-Szora A Floareş A Badea R Baciut G. Solid Parotid Tumors: An Individual and Integrative Analysis of Various Ultrasonographic Criteria. A Prospective and Observational Study. Med Ultrason (2013) 15(4):289–98. 10.11152/mu.2013.2066.154.afb2 24286093

[38] Fischer DR Baltzer P Malich A Wurdinger S Freesmeyer MG Marx C. Is the “Blooming Sign” A Promising Additional Tool to Determine Malignancy in MR Mammography? Eur Radiol (2004) 14(3):394–401. 10.1007/s00330-003-2055-9 14517688

[39] Stennert E Guntinas-Lichius O Klussmann JP Arnold G. Histopathology of Pleomorphic Adenoma in the Parotid Gland: A Prospective Unselected Series of 100 Cases. Laryngoscope (2001) 111(12):2195–200. 10.1097/00005537-200112000-00024 11802025

[40] Woo SH Choi DS Kim JP Park JJ Joo YH Chung PS. Two-Phase Computed Tomography Study of Warthin Tumor of Parotid Gland: Differentiation From Other Parotid Gland Tumors and Its Pathologic Explanation. J Comput Assist Tomogr (2013) 37(4):518–24. 10.1097/RCT.0b013e31828aede8 23863526

[41] Cingi E Ilcayto R Keã§Eli M Kalfa A. Papillary Cystadenoma Lymphomatosum (Warthin’s Tumor). J Indian Med Assoc (1955) 25(1):625–9.

[42] Jang M Park D Lee SR Hahm CK Kim Y Kim Y. Basal Cell Adenoma in the Parotid Gland: CT and MR Findings. AJNR Am J Neuroradiol (2004) 25(4):631–5. PMC7975606 15090357

[43] Naeim F Forsberg MI Waisman J Coulson WF. Mixed Tumors of the Salivary Glands. Growth Pattern and Recurrence. Arch Pathol Lab Med (1976) 100(5):271–5. 178290

[44] Lam PD Kuribayashi A Imaizumi A Sakamoto J Sumi Y Yoshino N. Differentiating Benign and Malignant Salivary Gland Tumours: Diagnostic Criteria and the Accuracy of Dynamic Contrast-Enhanced MRI With High Temporal Resolution. Br J Radiol (2015) 88(1049):20140685. 10.1259/bjr.20140685 25791568 PMC4628473

[45] Ikeda M Motoori K Hanazawa T Nagai Y Yamamoto S Ueda T. Warthin Tumor of the Parotid Gland: Diagnostic Value of MR Imaging With Histopathologic Correlation. AJNR Am J Neuroradiol (2004) 25(7):1256–62. PMC7976549 15313720

[46] Okahara M Kiyosue H Matsumoto S Hori Y Tanoue S Uchida D. Basal Cell Adenoma of the Parotid Gland: Mr Imaging Findings With Pathologic Correlation. AJNR Am J Neuroradiol (2006) 27(3):700–4. PMC7976949 16552019

[47] Kawata R Yoshimura K Lee K Araki M Takenaka H Tsuji M. Basal Cell Adenoma of the Parotid Gland: A Clinicopathological Study of Nine Cases–Basal Cell Adenoma Versus Pleomorphic Adenoma and Warthin’s tumor. Eur Arch Otorhinolaryngol (2010) 267(5):779–83. 10.1007/s00405-009-1139-9 19908055

