# Supplementary material for: Comparative Study of Qualitative and Quantitative Analyses of Contrast-Enhanced Ultrasound and the Diagnostic Value of B-Mode and Color Doppler for Common Benign Tumors in the Parotid Gland
Source: Front Oncol. 2021 Jul 7;11:669542. doi: 10.3389/fonc.2021.669542 (PMC8292955; doi:10.3389/fonc.2021.669542)
Supplement: Supplementary file 1 [file Table_1.docx]

**Supplementary information**

**Supplementary materials for Table 4**

**Multiple comparisons**

**Post Hoc Test:**

**Variable：PI, peak intensity (dB) (LSD method)**

| (I)group | (J)group | Standard error | *p* value | 95% confidence interval | |
| --- | --- | --- | --- | --- | --- |
| WT | PA | .34260 | .041 | .0291 | 1.3874 |
|  | BCA | .42818 | .001 | -2.3278 | -.6302 |
| PA | WT | .34260 | .041 | -1.3874 | -.0291 |
|  | BCA | .39581 | .000 | -2.9719 | -1.4026 |
| BCA | WT | .42818 | .001 | .6302 | 2.3278 |
|  | PA | .39581 | .000 | 1.4026 | 2.9719 |

P < 0.050 was considered to be statistically significant.

**Variable：MTT, mean transit time (Tamhane’s T2 method)**

| (I)group | (J)group | Standard error | *p* value | 95% confidence interval | |
| --- | --- | --- | --- | --- | --- |
| WT | PA | 4.40289 | .000 | 16.2218 | 38.0917 |
|  | BCA | 4.40495 | .000 | 12.4017 | 34.3002 |
| PA | WT | 4.40289 | .000 | -38.0917 | -16.2218 |
|  | BCA | 2.27915 | .271 | -9.4039 | 1.7923 |
| BCA | WT | 4.40495 | .000 | -34.3002 | -12.4017 |
|  | PA | 2.27915 | .271 | -1.7923 | 9.4039 |

P < 0.050 was considered to be statistically significant.

**Variable：AUC, area under curve (Tamhane’s T2 method)**

| (I)group | (J)group | Standard error | *p* value | 95% confidence interval | |
| --- | --- | --- | --- | --- | --- |
| WT | PA | 37.98021 | .009 | 23.9950 | 211.1606 |
|  | BCA | 37.42922 | .965 | -108.4589 | 76.7062 |
| PA | WT | 37.98021 | .009 | -211.1606 | -23.9950 |
|  | BCA | 25.64553 | .000 | -196.3494 | -70.5590 |
| BCA | WT | 37.42922 | .965 | -76.7062 | 108.4589 |
|  | PA | 25.64553 | .000 | 70.5590 | 196.3494 |

P < 0.050 was considered to be statistically significant.

**Variable：HT, time from peak to one half (Tamhane’s T2 method)**

| (I)group | (J)group | Standard error | *p* value | 95% confidence interval | |
| --- | --- | --- | --- | --- | --- |
| WT | PA | 4.30776 | .000 | 9.1622 | 30.4215 |
|  | BCA | 4.90205 | .000 | 8.0694 | 32.2275 |
| PA | WT | 4.30776 | .000 | -30.4215 | -9.1622 |
|  | BCA | 3.69404 | 1.000 | -8.8495 | 9.5626 |
| BCA | WT | 4.90205 | .000 | -32.2275 | -8.0694 |
|  | PA | 3.69404 | 1.000 | -9.5626 | 8.8495 |

P < 0.050 was considered to be statistically significant.

**Supplementary materials for Figure 6**

**Legend:**

**Fig. 6.** Receiver operating characteristic (ROC) curve analysis. (A) regarding RS, ROC curve for WT and BCA tumors (P < 0.001, AUC = 0.836); (B) regarding MTT, ROC curve for WT and PA tumors (P < 0.001 and AUC = 0.841); (C) regarding HT, ROC curve for WT and PA tumors (P < 0.001 and AUC = 0.750, respectively); (D) regarding PI; ROC curve for PA and BCA tumors (P < 0.001 and AUC = 0.871).

**Case Processing Summary**

1. **Group**

| Positive^a^ | 22 |
| --- | --- |
| Negative | 34 |

1. The positive actual state is BCA.

**Area Under the Curve**

**Test results variable: rising slope (RS) (dB/s)**

|  |  | Asymptopic | Asymptopic 95% Confidence Interval | |
| --- | --- | --- | --- | --- |
| Area | Std. Error^a^ | Sig.^b^ | Lower bound | Upper bound |
| 0.836 | 0.062 | 0.000 | 0.715 | 0.958 |

1. Under the nonparametric assumption
2. Null hypothesis: true area = 0.5
3. **Group**

| Positive^a^ | 34 |
| --- | --- |
| Negative | 54 |

1. The positive actual state is WT.

**Area Under the Curve**

**Test results variable: mean transit time (MTT) (s)**

|  |  | Asymptopic | Asymptopic 95% Confidence Interval | |
| --- | --- | --- | --- | --- |
| Area | Std. Error^a^ | Sig.^b^ | Lower bound | Upper bound |
| 0.841 | 0.043 | 0.000 | 0.756 | 0.926 |

1. Under the nonparametric assumption
2. Null hypothesis: true area = 0.5
3. **Group**

| Positive^a^ | 34 |
| --- | --- |
| Negative | 54 |

1. The positive actual state is WT.

**Area Under the Curve**

**Test results variable: time from peak to one half (HT) (s)**

|  |  | Asymptopic | Asymptopic 95% Confidence Interval | |
| --- | --- | --- | --- | --- |
| Area | Std. Error^a^ | Sig.^b^ | Lower bound | Upper bound |
| 0.750 | 0.054 | 0.000 | 0.645 | 0.855 |

1. Under the nonparametric assumption
2. Null hypothesis: true area = 0.5
3. **Group**

| Positive^a^ | 22 |
| --- | --- |
| Negative | 54 |

1. The positive actual state is BCA.

**Area Under the Curve**

**Test results variable: peak intensity (PI) (dB)**

|  |  | Asymptopic | Asymptopic 95% Confidence Interval | |
| --- | --- | --- | --- | --- |
| Area | Std. Error^a^ | Sig.^b^ | Lower bound | Upper bound |
| 0.871 | 0.041 | 0.000 | 0.791 | 0.951 |

1. Under the nonparametric assumption
2. Null hypothesis: true area = 0.5
